# Posterior Reversible Encephalopathy Syndrome (PRES) Following Fruquintinib in a Normotensive Patient With Metastatic Colorectal Cancer: A Case Report

**DOI:** 10.7759/cureus.97598

**Published:** 2025-11-23

**Authors:** Rawan ElManfalouty, Ahmed Holayel, Ian Russell, Kalena Marti

**Affiliations:** 1 Oncology, The Christie NHS Foundation Trust, Manchester, GBR; 2 Oncology, Maidstone and Tunbridge Wells NHS Trust, Kent, GBR; 3 Radiology, The Christie NHS Foundation Trust, Manchester, GBR

**Keywords:** chemotherapy-related toxicity, metastatic colorectal cancer (mcrc), neurotoxic, posterior reversible encephalopathy syndrome (pres), vegfr inhibitor

## Abstract

We report a rare case of posterior reversible encephalopathy syndrome (PRES) following fruquintinib treatment in a 71-year-old female with metastatic colorectal adenocarcinoma harbouring a KRAS G12D mutation. The patient had previously undergone multiple lines of chemotherapy and surgical resections with stable disease before receiving fruquintinib under compassionate access. After two cycles, she developed headaches and acute encephalopathy. MRI confirmed PRES without associated hypertension. Fruquintinib was discontinued, and the patient made a full neurological recovery. This case highlights a rare but significant toxicity of anti-vascular endothelial growth factor (VEGF) therapy in the colorectal cancer setting and underscores the importance of early radiological evaluation in suspected PRES.

## Introduction

Colorectal cancer remains a major global health burden and continues to be one of the leading causes of cancer-related mortality worldwide [1]. The management of metastatic colorectal cancer (mCRC) has evolved significantly with the introduction of targeted therapies that inhibit specific molecular pathways involved in tumour angiogenesis and proliferation [2].

Fruquintinib is an oral, highly selective small-molecule inhibitor of vascular endothelial growth factor receptors (VEGFR) 1, 2, and 3 [3]. It has demonstrated clinical benefit in patients with refractory mCRC who have progressed after standard chemotherapy regimens, by effectively inhibiting tumour angiogenesis [3]. Although fruquintinib is generally well tolerated, it shares with other anti-VEGF agents the potential to cause vascular complications arising from endothelial injury and compromise of the blood-brain barrier.

Posterior reversible encephalopathy syndrome (PRES) is a rare but potentially serious neurological complication that has been linked to VEGF inhibition [4]. To our knowledge, PRES associated with fruquintinib has been rarely reported, emphasizing the need for clinical awareness. It is characterized by clinical features such as headache, seizures, visual disturbances, and altered mental status, along with radiological findings of vasogenic oedema, typically affecting the parieto-occipital regions of the brain [4].

Here, we present a case of PRES associated with fruquintinib therapy in a patient with KRAS-mutant metastatic colorectal cancer. To our knowledge, published data on this association are extremely limited, and this report aims to contribute to the existing literature by highlighting the importance of awareness and prompt recognition of this rare adverse effect.

## Case presentation

A 71-year-old woman with a history of hypothyroidism and well-controlled asthma was diagnosed in early 2021 with right-sided colorectal adenocarcinoma with synchronous liver metastases involving five hepatic segments. Molecular profiling confirmed a KRAS G12D mutation with microsatellite-stable (MSS) status, and wild-type NRAS, BRAF, and PIK3CA.

She underwent a right hemicolectomy in February 2021 with histology showing pT4a pN1b M1 and R0 resection. This was followed by 12 cycles of adjuvant FOLFOX chemotherapy (5-fluorouracil and oxaliplatin), completed in November 2021. In January 2022, a right hemi-hepatectomy was performed with resection of six viable liver metastases. No left lobe lesions were identified.

Despite initial disease control, the patient developed unresectable hepatic recurrence. She was treated with second-line FOLFIRI (5-fluorouracil, folinic acid and irinotecan) plus bevacizumab until August 2022, followed by third-line TAS-102 (trifluridine and tipiracil). Disease progression prompted initiation of regorafenib in September 2023, which was discontinued in January 2024 due to progression. Rechallenge with FOLFIRI plus bevacizumab began in February 2024, followed by fruquintinib (5mg) under compassionate access in December 2024.

In January 2025, after one cycle, the patient reported mild headaches. Following the second cycle (February 2025), she presented to Salford Royal Hospital with severe headache and altered mental status. Neurological examination revealed transient encephalopathy without focal deficits. Blood pressure remained within normal limits (around 130/80). On review she had normal renal functions and was not on any neurotoxic agents. 

**Figure 1:**
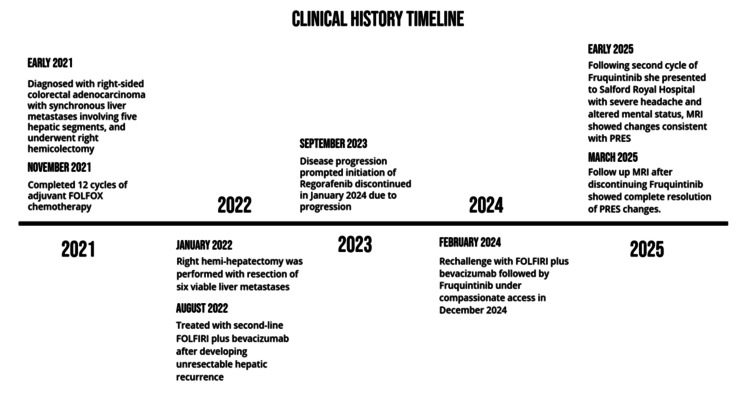
Clinical history timeline PRES: posterior reversible encephalopathy syndrome

A summary of the timeline is illustrated below in Figure 1.

**Figure 2:**
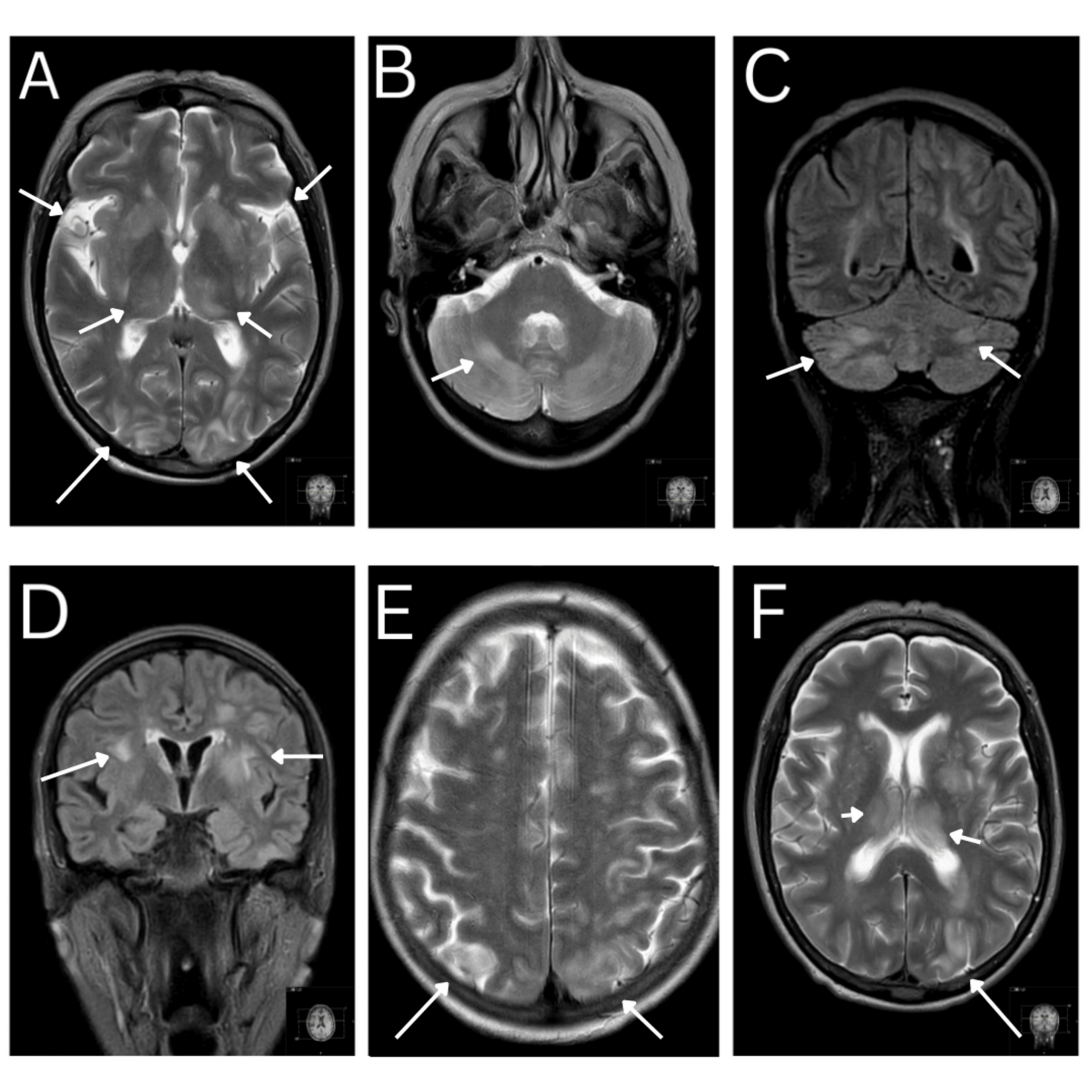
High T2 signals within cerebrum and cerebellum consistent with posterior reversible encephalopathy syndrome (PRES) A: Axial MRI head showing high T2 signal within the subcortical white matter of basal ganglia, both occipital lobes and thalami B: Axial MRI head showing high T2 signal within the subcortical white matter of cerebellar hemispheres C: Coronal MRI head showing high T2 fluid-attenuated inversion recovery (FLAIR) signals throughout cerebellum D: Coronal MRI head showing high T2 FLAIR signal within the subcortical white matter of left inferior temporal lobe and basal ganglia E: Axial MRI head showing posterior high T2 signal within occipital lobe F: Axial MRI head showing high T2 signal within the left parietal lobe, basal ganglia and thalami

MRI brain showed bilateral subcortical T2/fluid-attenuated inversion recovery (FLAIR) hyperintensities in occipital, parietal, and temporal lobes, cerebellar hemispheres, and basal ganglia, with no diffusion restriction or enhancement - findings consistent with PRES. There was no evidence of metastasis (Figure 2). 

**Figure 3:**
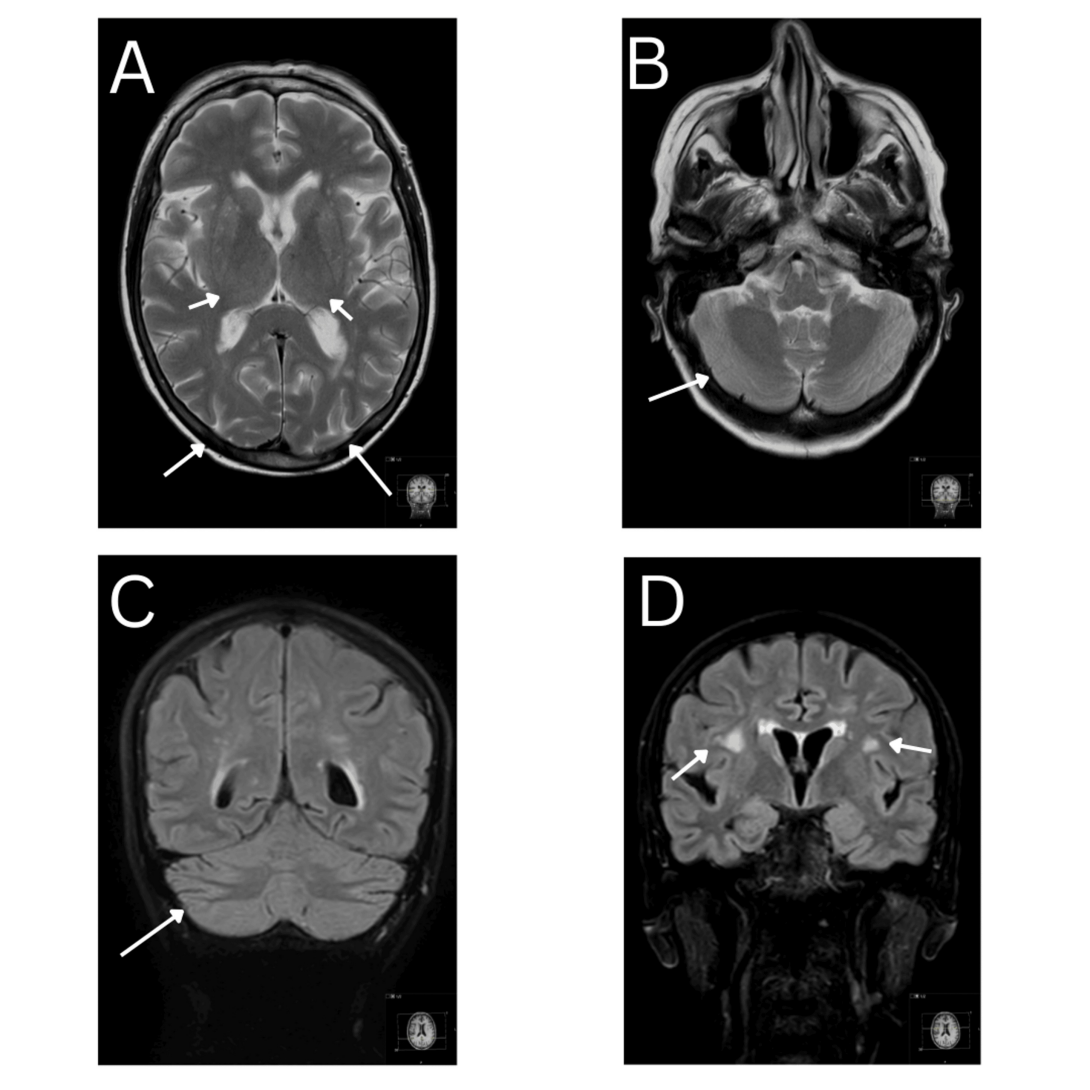
Resolution of changes in cerebrum and cerebellum confirming posterior reversible encephalopathy syndrome (PRES) A: Axial MRI head showing resolution of signal changes within the subcortical white matter of basal ganglia, both occipital lobes and thalami B: Axial MRI head showing resolution of signal changes in cerebellum C: Coronal MRI head showing resolution of changes in cerebellum on fluid-attenuated inversion recovery (FLAIR) MRI D: Coronal MRI head showing resolution of changes in inferior temporal lobe and basal ganglia on FLAIR MRI

Fruquintinib was discontinued, and the patient’s symptoms resolved spontaneously. A follow-up MRI in March 2025 showed complete radiological resolution of PRES changes, confirming the diagnosis (Figure 3). 

## Discussion

PRES is a clinico-radiological entity that was first described in 1996 in a series of 15 patients with acute neurological symptoms including headache, seizures, visual disturbances, and other focal neurological deficits. PRES is associated with a wide array of clinical presentations including headaches, focal neurological deficits, seizures, visual disturbances, and encephalopathy. The severity and acuity of clinical symptoms vary, although typically occur with rapid onset. The diagnosis of PRES is typically made with magnetic resonance imaging (MRI) of the brain. Imaging characteristically shows focal regions of symmetric hyperintensities on T2-weighted studies most commonly in the parietal and occipital lobes, followed by the frontal lobes and the cerebellum. It can also involve basal ganglia, thalami, deep white matter, brainstem, and even spinal cord in rare cases [5]. Rarely, PRES can present without prominent encephalopathy, with headache (and perhaps seizures or visual changes) as the only presenting symptom [6].

Fruquintinib is a novel oral small-molecule tyrosine kinase inhibitor (TKI) that selectively targets vascular endothelial growth factor receptors VEGFR-1, VEGFR-2, and VEGFR-3 [7]. Its primary anti-tumour mechanism lies in inhibition of angiogenesis, a process critical for tumour growth and metastasis. VEGF signaling promotes endothelial cell proliferation, migration, and survival, and contributes to the integrity of the blood-brain barrier (BBB) [8]. 

By blocking VEGFR-mediated signaling, VEGF inhibitors can disrupt vascular homeostasis and endothelial function, increasing vascular permeability and potentially leading to leakage of fluid into the interstitial and cerebral spaces. This can culminate in vasogenic edema, a hallmark of PRES. Additionally, VEGF inhibition can impair nitric oxide (NO) production and autoregulation of cerebral blood flow, making the brain more susceptible to fluctuations in systemic blood pressure-even in the absence of overt hypertension. Indeed, endothelial injury independent of hypertension is increasingly recognised, indicating that PRES can arise solely from endothelial dysfunction without the classical severe hypertensive surge [9].

Although PRES is more commonly reported with VEGF-targeting monoclonal antibodies such as bevacizumab, small-molecule TKIs like sorafenib, sunitinib, and regorafenib have also been implicated [10].Fruquintinib shares a similar target profile and, although rarely reported, has now been associated with PRES in a small number of published cases. 

Our case further supports this link. The patient developed classical imaging features of PRES involving both supra- and infratentorial regions shortly after completing two cycles of fruquintinib. Notably, she remained normotensive, reinforcing the hypothesis that endothelial dysfunction due to VEGF inhibition alone - independent of hypertension - can be sufficient to trigger PRES. Her symptoms resolved spontaneously after the drug was withdrawn, and follow-up MRI confirmed complete resolution of imaging abnormalities, fulfilling the criteria for PRES.

It is important to recognize that fruquintinib is increasingly used in later-line settings for metastatic colorectal cancer, particularly in patients with limited remaining treatment options. As its clinical use expands, oncologists and neurologists should maintain a high index of suspicion for PRES in any patient presenting with acute neurological symptoms while on VEGF-targeted therapy - even in the absence of hypertension or other classic risk factors.

When confronted with acute neurological symptoms such as headache, seizures, and visual disturbances in a cancer patient on anti-VEGF therapy, it is vital to exclude other potential causes before attributing findings to PRES. Important considerations include acute ischaemic or haemorrhagic stroke; brain metastases which may present with new seizures, focal deficits, and MRI findings of enhancing lesions; and central nervous system infection or encephalitis which may mimic PRES clinically (altered mentation, seizures) and demonstrate diffusion-restriction or contrast enhancement.

In our case, the imaging pattern (described above), the absence of enhancing metastatic lesions, and the full radiological and clinical reversal after drug withdrawal all support PRES over these alternatives.

## Conclusions

The findings of this study underscore that PRES may develop in patients receiving fruquintinib, even in the absence of hypertension. Early recognition remains crucial, as prompt neuroimaging and immediate withdrawal of the offending agent are typically associated with complete neurological recovery. In clinical practice, incorporating baseline and periodic blood pressure monitoring together with routine neurological assessment may facilitate timely detection of PRES. Vigilance for new or unexplained neurological symptoms in patients treated with fruquintinib is therefore strongly recommended to ensure early diagnosis and optimal outcomes.
